# A Plea for the Woman Physician

**Published:** 1915-01

**Authors:** 


**A Monthly Review of Medicine and Surgery**

EDITOR AND PUBLISHER, DR. A. L. BENEDICT, 228 Summer St., corner of Elmwood Ave., Buffalo, (Address for all communications. Please make personal and telephone calls before 1 P. M.)

Third, in age, on the western continent. Independent, but supports professional organization. News limited mainly to Western N. Y. and Penn.

Subscription $2.00 a year; $3.00 for two years in advance. Changes and errors in address or failure or duplication of delivery, should be reported immediately.

Yearly Volume 70. JANUARY, 1915. No. 6

### A Plea For The Woman Physician.

We have read, with some indignation, an article in a medical journal which seeks to excite pity for the woman doctor—she encounters so much prejudice; has no opportunity to make acquaintances in clubs, etc., as men have; ages so much more rapidly than her male classmates; cannot get a consultation practice; has no chance at hospitals; male physicians will not be friendly to her; work is too hard for her; is becoming extinct because of the lack of demand for her services; needs the vote to improve her sad lot. A similar article received wide publicity in a literary magazine some months ago. The latter was prepared by a professional writer and was based on more or less authentic interviews. The former was anonymous but rather implies that it was written by a woman doctor though the general style and viewpoint make us sceptic on this point.

To ask or to receive pity is humiliating. To offer it unasked is insulting, unless the fact that it is needed is beyond question. To ask it without such substantiation in fact indicates a lack of self respect. We feel confident that the articles referred to, do not represent either actual fact typic of the status of woman physicians or the sentiments of women physicians themselves. That there is a deep rooted idea of sex characteristics as applying to the fitness of men and of women for certain lines of work, must be admitted. Unquestionably, this idea does reduce the demand for women physicians much below that for men and imposes some obstacles to consultation practice, hospital appointment, etc. But, relatively to the percentages of the sexes in our profession, we question whether the demand for women is not greater than that for men. There is certainly, a far greater number of salaried positions open to women physicians in proportion to their number, than to men. That the number of women entering medical practice has declined, relatively and absolutely, is not so much due to any essential difficulty in finding support as to that nice sense of conditions commonly termed intuition, which women possess to a higher degree than men and which makes them realize, not so much the lack of demand for women physicians as for physicians in general. It must also be frankly admitted that social conditions have not altered so much in the last three generations as to compel the average woman of the average class which supplies the medical and other professions, to engage in direct gainful occupation, to the same extent as it does men. At the same time, nursing, social service and business have opened wide fields to women and those naturally compete to a greater degree than for men, for such women as are compelled or desire to enter gainful occupations.

Quite aside from the subject under discussion, we are sceptic as to the existence of communities in this country where there are not ample opportunities for a woman in any line of activity—or of domestic passivity—to meet other women in social, religious, philanthropic, literary and other societies. So far as our observation goes, such opportunities to secure acquaintance are far more ample for women than for men. And we question seriously whether, for men or women, acquaintance of this sort goes very far toward building up a practice.

That the practice of medicine is fatiguing, cannot be denied. From the sentimental side, we were at one time inclined to consider that this fact really did constitute a bar to the entrance of women into medicine. But, with longer experience, it has seemed to us that this idea has been verified. Every one ages and is finally driven out of work or dies in the harness. Allowing for the usual slightly greater age at which women enter medicine or any other occupation in which they compete with men on equal terms, it has not seemed to us that women age more rapidly than men or that the average life time of women in medicine is less than that in domestic life.

It has never seemed to us that prejudice has existed against the woman physician in her own profession, except sporadically, or that the friendship and good fellowship which ought to permeate the medical profession has been denied to women except in the sense that sexless friendship across sex lines is always somewhat difficult and that the social life of any body of human beings in which one sex greatly preponderates, tends to assume the form of a one-sex function from which the sex which is in the minority is more or less excluded, not from deliberate antagonism but because, in social matters we naturally follow social precedents.

Western New York is important historically in regard to the admission of women to the medical profession. Years ago, the Geneva Medical College, after provisional favorable vote of the faculty, placed the matter before the student body, which pledged itself to a cordial reception and courteous treatment of a woman student. The medical schools of this region, in which we are particularly interested, have followed this precedent ever since. There have been, of course, manifestations of individual prejudice but even these have been outgrown in most instances. There have been manifestations of a spirit of cameraderie which have been too literal fulfillments of the claim made by the writer first referred to in this article. There have been instances of personal prejudice unfavorable to the admission of women to the medical profession or any other change of time-honored institutions but, in the great majority of instances, in a broad spirit of courtesy to opposing views held by other individuals. A year or two ago, the women physicians of this region, at a social gathering following a scientific session, expressed an appreciation of the friendship of their brother M. D.'s and of the mutual good will existing between the sexes in medicine which we believe to be far more typic of the general conditions than the pessimistic article referred to.

In medicine, we are inclined to rely mainly on the clinical test. There are undoubtedly women as there are men in the profession, who have not prospered, who have overworked and who have been underpaid and under appreciated. But, so far as we can judge from the majority of the women in medicine in the cities and towns that we represent, they are living contradictions of the literary complaints to which we have alluded. They do not need or desire pity from anyone.

This is, of course, written from a man's standpoint. What have the ladies to say on the matter?

